# O-Antigen Diversification Masks Identification of Highly Pathogenic Shiga Toxin-Producing Escherichia coli O104:H4-Like Strains

**DOI:** 10.1128/spectrum.00987-23

**Published:** 2023-05-22

**Authors:** Christina Lang, Angelika Fruth, Ian W. Campbell, Claire Jenkins, Peyton Smith, Nancy Strockbine, François-Xavier Weill, Ulrich Nübel, Yonatan H. Grad, Matthew K. Waldor, Antje Flieger

**Affiliations:** a Division of Enteropathogenic Bacteria and Legionella, National Reference Centre for Salmonella and Other Enteric Bacterial Pathogens, Robert Koch Institut, Wernigerode, Germany; b Department of Microbiology, Division of Infectious Diseases, Brigham and Women’s Hospital, Harvard Medical School, Boston, Massachusetts, USA; c Gastro and Food Safety (One Health) Division, Health Security Agency, London, United Kingdom; d Division of Foodborne, Waterborne and Environmental Diseases, National Center for Emerging and Zoonotic Infectious Diseases, Centers for Disease Control and Prevention, Atlanta, Georgia, USA; e Institut Pasteur, Université Paris Cité, Unité des Bactéries Pathogènes Entériques, Paris, France; f Leibniz Institute DSMZ-German Collection of Microorganisms and Cell Cultures, Braunschweig, Germany; g German Center for Infection Research (DZIF), Partner Site Braunschweig-Hannover, Hannover, Germany; h Braunschweig Integrated Center of Systems Biology (BRICS), Technical University, Braunschweig, Germany; i Department of Immunology and Infectious Diseases, Harvard T. H. Chan School of Public Health, Boston, Massachusetts, USA; j Howard Hughes Medical Institute, Boston, Massachusetts, USA; Post Graduate Institute of Medical Education and Research

**Keywords:** O-antigen diversification, O104:H4, Shiga toxin-producing *E. coli*, phylogeny, risk profiling

## Abstract

Shiga toxin-producing Escherichia coli (STEC) can give rise to a range of clinical outcomes from diarrhea to the life-threatening systemic condition hemolytic-uremic syndrome (HUS). Although STEC O157:H7 is the serotype most frequently associated with HUS, a major outbreak of HUS occurred in 2011 in Germany and was caused by a rare serotype, STEC O104:H4. Prior to 2011 and since the outbreak, STEC O104:H4 strains have only rarely been associated with human infections. From 2012 to 2020, intensified STEC surveillance was performed in Germany where the subtyping of ~8,000 clinical isolates by molecular methods, including whole-genome sequencing, was carried out. A rare STEC serotype, O181:H4, associated with HUS was identified, and like the STEC O104:H4 outbreak strain, this strain belongs to sequence type 678 (ST678). Genomic and virulence comparisons revealed that the two strains are phylogenetically related and differ principally in the gene cluster encoding their respective lipopolysaccharide O-antigens but exhibit similar virulence phenotypes. In addition, five other serotypes belonging to ST678 from human clinical infection, such as OX13:H4, O127:H4, OgN-RKI9:H4, O131:H4, and O69:H4, were identified from diverse locations worldwide.

**IMPORTANCE** Our data suggest that the high-virulence ensemble of the STEC O104:H4 outbreak strain remains a global threat because genomically similar strains cause disease worldwide but that the horizontal acquisition of O-antigen gene clusters has diversified the O-antigens of strains belonging to ST678. Thus, the identification of these highly pathogenic strains is masked by diverse and rare O-antigens, thereby confounding the interpretation of their potential risk.

## INTRODUCTION

Shiga toxin-producing Escherichia coli (STEC) is a foodborne pathogen responsible for a range of clinical syndromes from diarrhea to the life-threatening systemic condition hemolytic-uremic syndrome (HUS), a triad of thrombotic microangiopathy, thrombocytopenia, and acute renal injury (1). Classical STEC strains include those of the pathovar enterohemorrhagic E. coli (EHEC), such as strains of serotype O157:H7, which, in addition to the Shiga toxin gene, contain the locus of enterocyte effacement (LEE) pathogenicity island coding for a virulence-associated type III secretion system and effectors (1). Historically, the classification of STEC strains into different serotypes has proven invaluable for epidemiology and risk profiling (2). In E. coli, the serotype is determined by a combination of O- and H-antigen types (see below), whereas the O group denotes solely the O-antigen. Globally, O157:H7 strains are most frequently associated with HUS, but furthermore, strains belonging to the O groups O26, O103, O111, and O145 have also been regularly linked to HUS development (2, 3). In addition, a very rare serotype gave rise to a major HUS outbreak in early summer 2011 when an O104:H4 strain caused more than 3,000 cases of diarrhea and 800 cases of HUS, including 54 fatalities, predominantly in Germany (4–6).

The O104:H4 outbreak strain encodes an exceptional set of virulence features (5–9). Like other HUS-associated strains, the strain produces Shiga toxin (Stx), specifically the Stx2a variant. But unlike most E. coli strains causing HUS, this strain belongs to enteroaggregative E. coli (EAEC), has acquired an *stx*_2a_-carrying phage, and lacks the LEE. EAEC strains characteristically harbor a plasmid (pAA) encoding aggregative adhesion fimbriae (AAF), in this case AAF of type I (AAF/I) (5, 7). AAF are responsible for bacterial autoaggregation, stacked-brick adhesion to host cells, and contribute to the inflammatory response (10, 11). Furthermore, the outbreak strain encodes the virulence-linked serine-protease autotransporters (SPATEs) SepA, SigA, and Pic and harbors an additional plasmid encoding an extended-spectrum β-lactamase (ESBL) of the CTX-M-15 type (5, 11, 12). The O104:H4 outbreak strain along with other O104:H4 strains all belong to multilocus sequence typing (MLST) sequence type 678 (ST678), and some possess *stx*, forming a distinct clade among EAEC strains (5, 7, 11). Despite the extensive outbreak in May/June of 2011 and the associated wide distribution of the strain in affected regions, intensified molecular surveillance uncovered relatively few O104:H4 cases in Germany after the outbreak dissipated by July 2011.

In E. coli, serotypes are determined by the composition of the lipopolysaccharide (LPS) O-antigen and the flagellar H-antigen, both of which are important surface features of microorganisms that shape pathogen-host interactions (13, 14). LPS forms a major structural component of the Gram-negative cell’s outer membrane, and its most distal part is the O-antigen. The O-antigen is subject to strong selection pressure and is one of the most variable components of the bacterial cell (13). Typically, in E. coli, the O-antigen consists of chains of repeating oligosaccharide subunits, usually composed of 2 to 7 sugars, often with additional chemical modifications (15); currently, 182 O groups and 53 flagellar antigen types have been described by phenotypic identification (14, 16). The genes encoding O-antigen biosynthesis are organized into clusters that are flanked by a colanic acid biosynthesis gene cluster (*wca* genes) and a histidine (*his*) biosynthesis operon (15). O-antigen biosynthesis gene clusters typically have a GC content (often <40%) lower than that of the backbone of the E. coli chromosome, which has an ~50% GC content (13, 17, 18). These differences suggest that O-antigen biosynthesis gene clusters are exchanged by lateral gene transfer, are under diversifying selection, and therefore are a hot spot of recombination (15, 19).

Despite its wide distribution in the affected areas, the near disappearance of E. coli O104:H4 in Germany after the large outbreak in 2011 was unanticipated. Here, we show that the high-virulence ensemble of the O104:H4 outbreak strain remains a threat but that O-antigen gene (OAG) exchange has cloaked the pathogen with several new O-antigens.

## RESULTS

### HUS-associated STEC O181:H4 strain 17-07187 shares a close phylogenetic relationship with and has virulence traits similar to those of the O104:H4 outbreak strain.

**FIG 1:**
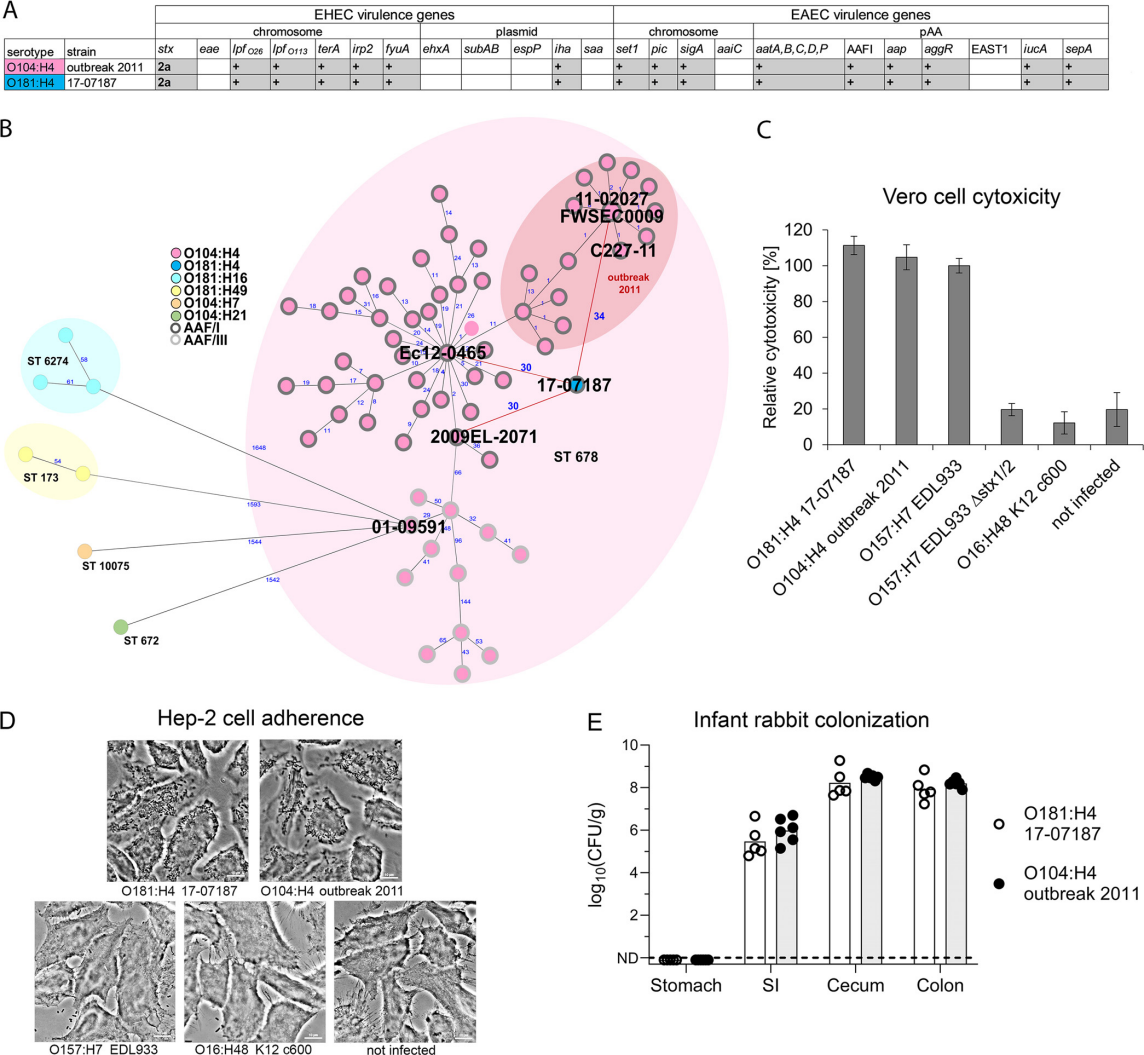
STEC O181:H4 17-07187 shares a close phylogenetic relationship with and has virulence traits similar to those of the O104:H4 outbreak strain. (A) Overlapping virulence gene profiles of the O104:H4 outbreak strain (FWSEC0009) and O181:H4 strain 17-07187. (B) A minimum-spanning tree of O181:H4 17-07187 and selected O104:H4 strains based on cgMLST involving 2,513 alleles confirms their close phylogenetic relationship. Strains representing the phylogenetic diversity of O104:H4 isolates from humans with respect to the isolation time (from 1998 to 2022) and location were selected. Only one cluster representative was used, except for the 2011 outbreak. O181:H4 17-07187 and O104:H4 strain 2009EL-2071, strain Ec12-0465, and outbreak strains from 2011 (including strains FWSEC0009, C227-11, and 11-02027) differ by only 30 to 34 alleles. An allele represents a variant form of a gene. Numbers in blue indicate allelic distances. Serotypes and AAF types are indicated in the key. For complete strain designations, see Fig. S1A in the supplemental material. (C and D) The cytotoxicity of O181:H4 17-07187 toward Vero cells (C) and the pattern of adherence to HEp-2 cells (D) are comparable to those of O104:H4 outbreak strain 11-02027. The results are representative of data from at least two additional experiments. EHEC O157:H7 EDL933 producing Stx1 and Stx2 served as a reference in the cytotoxicity assay and was set to 100%. The cytotoxicity results represent the means and standard deviations for triplicate samples (*n* = 3) and are representative of data from at least two additional experiments. (E) Intestinal colonization in infant rabbits inoculated with O104:H4 outbreak strain C227-11 (*n* = 6) or O181:H4 strain 17-07187 (*n* = 5). The CFU per gram of tissue denotes the concentration of bacteria recovered at 3 days postinoculation from homogenized intestinal tissues. Data points represent individual rabbits from two independent litters split between the two strains. Bars show the geometric means. SI, small intestine; ND, not detected.

After the large STEC O104:H4 outbreak in 2011, the German National Reference Centre for Salmonella and Other Bacterial Enteric Pathogens intensified STEC surveillance and analyzed ~8,000 clinical isolates primarily from diarrhea and HUS patients from 2012 to 2020. This strain collection included a stool sample isolate (17-07187) from a 6-year-old girl who had bloody diarrhea and HUS in December 2017. She had not traveled outside her home in Northwest Germany before becoming ill. Serotyping and whole-genome sequencing (WGS) revealed that the strain belonged to an unusual serotype, O181:H4, that had not been previously associated with HUS. The strain had *stx*_2a_ but lacked the LEE pathogenicity island (marker gene *eae*). Furthermore, the strain carried characteristic EAEC markers, including *aatA*, *aggR*, AAF/I genes, and the autotransporter protease genes *pic*, *sigA*, and *sepA* (Fig. 1A; see also Table S1 in the supplemental material).

MLST demonstrated that the 17-07187 isolate belonged to the same sequence type (ST678) as the O104:H4 outbreak strain (Table S2) (5, 7, 9). The EnteroBase core-genome MLST (cgMLST) scheme (2,513 genes) (20) confirmed the close genomic relationship of this isolate with a panel of O104:H4 strains (Fig. 1B and Fig. S1A). There were only 30 to 34 allelic distances (ADs) between this O181:H4 isolate and the O104:H4 outbreak strain (e.g., strain FWSEC0009, C227-11, or 11-02027) and O104:H4 clinical isolates from the Republic of Georgia in 2009 (2009EL-2071) and France in 2012 (Ec12-0465). In contrast, there were considerable ADs to STEC serotypes O181:H16 (ST6274), O181:H49 (ST173), O104:H21 (ST672), and O104:H7 (ST10075) isolated between 2012 and 2019 (ADs of >1,500) (Fig. 1B). Comparison of the virulence gene repertoires of the O181:H4 and the O104:H4 outbreak strains also strongly suggested that they rely on very similar virulence mechanisms (Fig. 1A). Indeed, both strains had comparable Stx-related cytotoxicity (Fig. 1C), exhibited a characteristic enteroaggregative adherence pattern (Fig. 1D), and colonized intestinal tissues, particularly the cecum and colon, similarly during *in vivo* infant rabbit infections (Fig. 1E). Thus, STEC O181:H4 and O104:H4 isolates share marked genomic similarity and virulence-associated genomic and phenotypic traits.

### The genomes of STEC O181:H4 strain 17-07187 and the O104:H4 outbreak strain differ mainly in their O-antigen gene clusters and mobile genetic elements.

**FIG 2:**
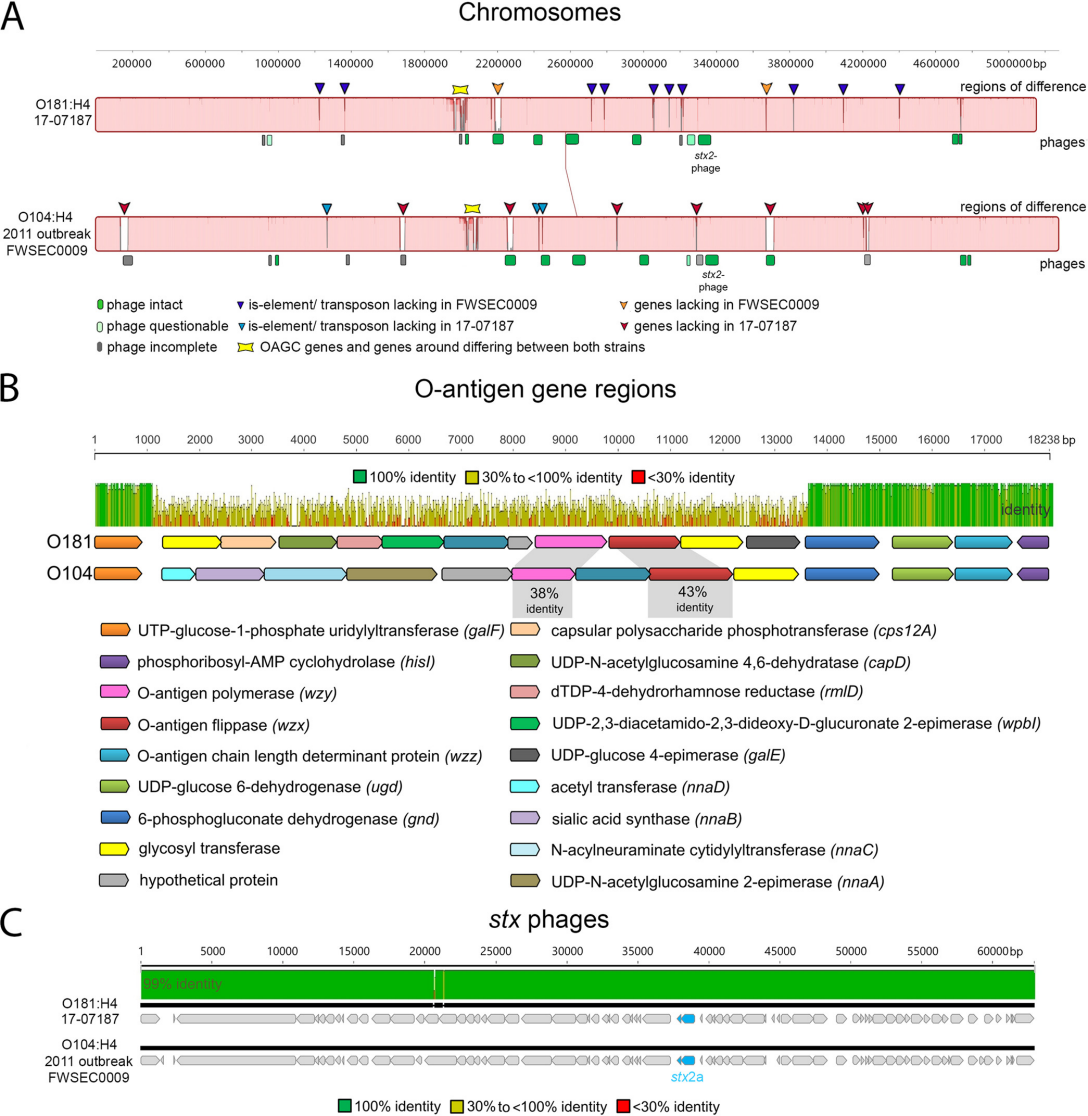
O181:H4 17-07187 and O104:H4 outbreak strain genomes differ mainly in OAGCs and mobile genetic elements. (A) Whole-chromosome MAUVE alignment of O181:H4 strain 17-07187 and O104:H4 outbreak strain FWSEC0009 highlighting mobile genetic elements and differences in phage regions, insertion sequence (IS)/transposon elements, and OAGC regions. (B) OAGCs of O181:H4 strain 17-07187 and O104:H4 outbreak strain FWSEC0009 are flanked by homologous upstream (*galF*) and downstream (*gnd* to *hisI*) regions. The MAFFT alignment shows that the regions between *galF* and *gnd* are very different. (C) MAFFT alignment of the *stx*_2a_-carrying phages in O181:H4 17-07187 and O104:H4 outbreak strain FWSEC0009 shows that they are very similar (99.9% nucleotide identity).

The chromosomes (without plasmids) of O181:H4 isolate 17-07187 and O104:H4 outbreak strain FWSEC0009 were very similar (~99.7% nucleotide identity in the core genome that is shared between these strains). The most striking difference between them was their respective O-antigen gene clusters (OAGCs) (Fig. 2A and B). Although these two clusters were both situated at the same location in the chromosome, between *galF* and *hisI* (Fig. 2B), their gene contents and organizations were very different. Furthermore, their respective GC contents, 36.8% for O181 and 37% for O104, differed from the chromosome GC content (~50.7%), highlighting the likely role of lateral gene transfer in driving OAGC exchange.

Fourteen potential prophage regions are present in the O181:H4 isolate, and 16 are present in the O104:H4 outbreak strain (Fig. 2A and Table S3). Eleven of the fourteen prophages exhibited substantial sequence identity (83 to 99.9%) to their O104:H4 counterparts, and importantly, the *stx*_2_-carrying prophages were nearly identical (~99.9% nucleotide identity) (Fig. 2C and Table S4). Both *stx* phages are inserted into the tryptophan repressor binding protein gene *wrbA*.

The genome of the O181:H4 isolate included three plasmids of ~81 kb, ~76 kb, and ~63 kb (Table S2). The largest O181:H4 plasmid (pEc17-07187-1) was an incompatibility group I1 (IncI1) plasmid that showed only partial homology to the ESBL resistance plasmid of the O104:H4 outbreak strain (~75% nucleotide identity) but was very similar (98% nucleotide identity) to pHUSEC41-1 of STEC O104:H4 strain HUSEC41 from 2001 (92 kb) (Fig. S1B) (3, 21). Both of these plasmids carry the *pilI-V* operon genes for thin pili. Unlike pHUSEC41-1, O181:H4 plasmid pEc17-07187-1 did not contain antibiotic resistance genes (Fig. S1B and Table S5). The O181:H4 plasmid (pEc17-07187-2/pAA) (76 kb) was very similar (99.8% nucleotide identity) to the O104:H4 outbreak strain plasmid pAA-EA11 and harbored virulence-associated loci, including *aggABCD*, which encode AAF/I (Fig. 1A, Fig. S1C, and Table S6) (7, 11). O181:H4 plasmid pEc17-07187-3 (63 kb) was not found in the O104:H4 outbreak strain; instead, it showed similarity to DHA plasmids of several enterobacteria coding for AmpC β-lactamase (22). However, unlike the Dhahran (DHA) plasmids, resistance determinants were not present in the O181:H4 isolate (Fig. S1D and Table S7). Together, these observations reveal the striking similarity of the chromosomes of the O181:H4 isolate and the O104:H4 outbreak strains and that their chief differences are confined to hot spots of recombination, i.e., their OAGCs, and to mobile genetic elements, particularly their plasmids.

### Additional recent global isolates of serotype O181:H4 and five other O groups belong to ST678.

**FIG 3:**
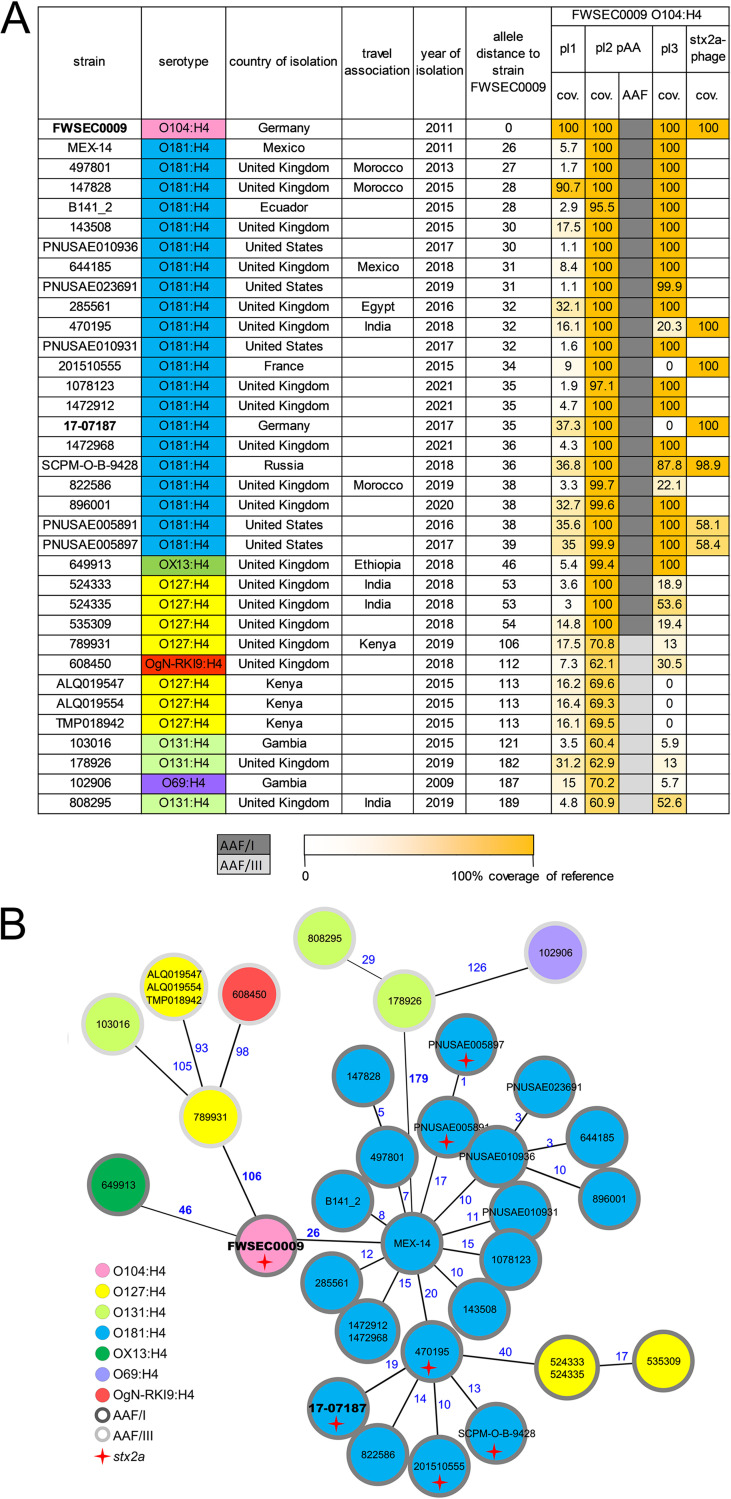
Recent global isolates of serotype O181:H4 and five other O groups belong to ST678. (A) Summary of serotypes, isolation dates, and relatedness of the genomes (cgMLST-based allelic distances) and mobile genetic elements (plasmids and *stx* phages) of the 34 non-O104:H4 ST678 (ST12598 and ST12610) clinical strains (found on EnteroBase) compared to O104:H4 outbreak strain FSWEC0009. (B) Serotypes of the non-O104:H4 ST678 strains and O104:H4 outbreak strain FSWEC0009 in a minimum-spanning tree based on cgMLST. Numbers in blue indicate allelic distances. Although phylogenetically very close to ST678 strains, O181:H4 strains 1472912 and 1472968 in fact belong to ST12610 due to a point mutation in *icd*, and OgN-RKI9:H4 strain 608450 belongs to ST12598 due to a point mutation in *recA*.

Next, we identified 158 genomes belonging to ST678 in EnteroBase (20), which contains ~202,200 E. coli genomes (as of 11 April 2022). For a subset, serotype identification was not available, and for these cases, the serotype was predicted based on the available genomic information (16). One hundred twenty-three of the ST678 strains were of O104:H4; however, 18 additional O181:H4 genomes of ST678 were found. Furthermore, 7 O127:H4 genomes, 3 O131:H4 genomes, and 1 genome each of O69:H4 and OX13:H4 were identified (Fig. 3A and Table S1). We categorized these as non-O104:H4 ST678 strains. Additionally, based on the close phylogenetic relationship and the single difference in MLST alleles, we added three non-ST678 strains to the non-O104:H4 ST678 category: two O181:H4 strains (1472912 and 1472968) and one strain of the new and provisionally assigned genoserotype OgN-RKI9:H4 (strain 608450) (Fig. 3A and Table S1).

cgMLST confirmed the close genetic relationship (AD of ~50) between AAF/I gene-positive O104:H4 strains, including the outbreak strain (FWSEC0009), and the 21 O181:H4 strains, the OX13:H4 strain, and 3 of the 7 O127:H4 strains (Fig. 3A and B). All of these strains showed AAF/I, which were also found in the 2011 outbreak strain (5, 7). Nine strains (four of O127:H4, three of O131:H4, and one each of O69:H4 and OgN-RKI9:H4) had higher ADs of up to 189. In contrast, these nine strains harbored AAF/III genes (Fig. 3A and B). The 25 AAF/I-positive non-O104:H4 ST678 strains were all isolated during or after 2011, the majority were associated with diarrheal disease, and a subset of 6 O181:H4 strains harbored *stx*_2a_ (Fig. 3A and B, Table S1, and Fig. S2A). Interestingly, four of these six genomes (including 17-07187 from Germany) shared an *stx* phage very similar to that of the O104:H4 outbreak strain, but two of the six prophages were more distinct (Fig. 3A). AAF/I-positive strains shared regions with higher relatedness than those positive for AAF/III with the pAA plasmid of the 2011 outbreak strain (Fig. 3A).

The virulence gene profiles of the 34 non-O104 ST678 strains were generally similar to that of the O104:H4 outbreak strain; however, there were a few differences. Specifically, *sepA* was found exclusively in AAF/I-positive strains, and EAST1 was present in all AAF/III-positive strains and only three of the AAF/I-positive isolates (Table S8).

The 34 non-O104:H4 ST678 strains were isolated in countries of Europe, Africa, and North and South America. In addition, several were from individuals with a travel history that might link these to East Asia (Fig. 3A and Fig. S2B and C). Together, these observations show that ST678 E. coli strains are found among seven different E. coli serotypes that have been linked to diarrheal disease on several continents.

### Phylogenomic analyses of ST678 E. coli strains suggest the occurrence of multiple O-antigen gene exchange events.

**FIG 4:**
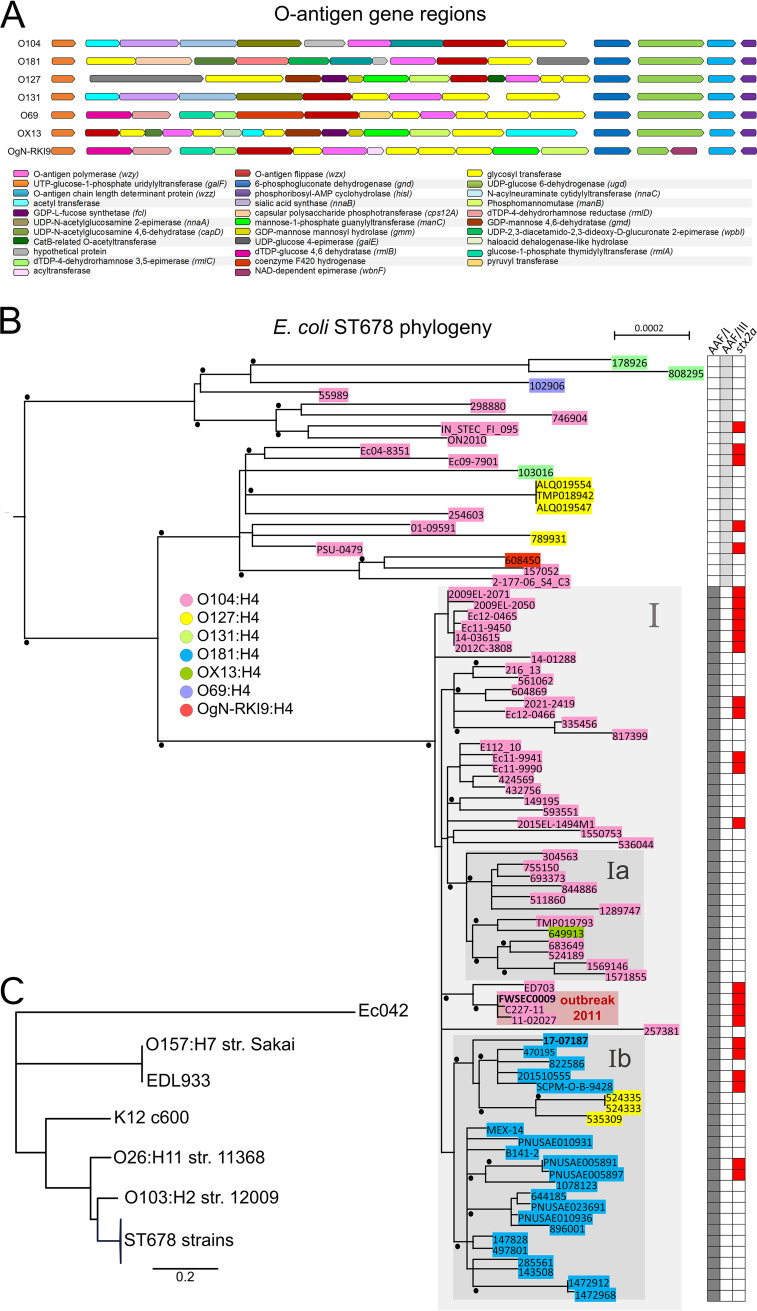
O-antigen gene clusters and phylogeny within E. coli ST678. (A) O-antigen gene clusters in ST678 strains differ in their gene contents and organizations, yet they are flanked by homologous regions starting upstream of *galF* and downstream of *gnd* to *hisI*, respectively. (B) Maximum likelihood phylogenetic tree generated by PhyML based on a recombination-corrected alignment of genome-wide polymorphic sites. Strains selected for representation in Fig. 1B and Fig. 3 were combined, and O104:H4 2011 outbreak strain FWSEC0009 served as a reference for read mapping. The tree was rooted with an outgroup consisting of E. coli strains K-12 c600, Ec042, and EDL933; O157:H7 strain Sakai; O26:H11 strain 11368; and O103:H2 strain 12009. Genome sequences from outgroup E. coli isolates had been included in the phylogenetic analysis and for clarity are not shown in panel B (large phylogenetic distances and associated limited resolution of the clades of interest) but are shown in panel C. Bootstrap values of >90% are indicated as black dots. Serotypes are indicated in the key. The heatmap on the right indicates the presence (filled boxes) or absence (white boxes) of AFF/I, AFF/III, and *stx*_2a_ genes. The scale bar refers to a phylogenetic distance of 0.0002 nucleotide substitutions per site. Gray boxes indicate clades. The box in light red highlights the 2011 outbreak strains. (C) Maximum likelihood phylogenetic tree with additional outgroup E. coli isolates and strains represented in panel B as a collapsed tree branch designated ST678 strains.

The OAGCs encoding the six O groups in the 34 non-O104:H4 ST678 strains are found at the same chromosomal location as the 2011 O104:H4 outbreak strain but are composed of largely disparate genes (Fig. 4A). These clusters also have a GC content (36.8 to 42.1%) distinct from that of the backbone genome (50.5 to 50.7%), suggesting that they were acquired by horizontal gene exchange. To explore the phylogenetic relationships among the 34 non-O104:H4 ST678 strains and a set of O104:H4 strains, their shared single nucleotide polymorphisms (SNPs) were analyzed using O104:H4 outbreak strain FWSEC0009 as a reference. A positive correlation (*R* = 0.81; *R*^2^ = 0,66) was found between the isolation time and genetic divergence (Fig. S2D), which supports that mutations have accumulated in a clocklike fashion without notable outliers. Phylogenetic analysis shows that AAF/I-positive strains within ST678 are closely related to each other, clustering into clade I, whereas AAF/III-positive strains are more diverse and form more basal branches in a rooted maximum likelihood phylogenetic tree (Fig. 4B). This structure of the phylogeny suggests that clade I strains were derived from an AAF/III-positive precursor. Within clade I, subclades Ia and Ib contain some non-O104:H4 strains. Clade Ia is composed of O104:H4 nonoutbreak strains isolated from 2015 to 2021 in the United Kingdom and Kenya and the 2018 OX13:H4 strain that was associated with travel to Ethiopia. Clade Ib contains all 21 O181:H4 strains and 3 O127:H4 strains isolated from diverse continents from 2011 to 2021. Since the majority of the ST678 strains are of serotype O104:H4, including basal phylogenetic branches and most isolates in clade I (Fig. 4B), it is parsimonious to assume that subclade Ib emerged from an O104:H4 predecessor by replacing O104 antigen genes with O181 antigen genes. Similarly, our phylogenetic analysis suggests that O127:H4 strains within clade Ib arose from an O181:H4 precursor. Together, these observations indicate that OAGC exchange has occurred repeatedly within ST678 strains.

## DISCUSSION

The E. coli O104:H4 outbreak in Germany in the early summer of 2011 was a public health emergency; however, this serotype was rarely isolated as a cause of HUS after the epidemic subsided. Nevertheless, our findings suggest that the unusual set of virulence factors that characterized this Shiga toxin 2-producing EAEC strain remains a threat to human health. Serotype conversion has cloaked this highly virulent genotype with several O-antigens, including O181, O127, and OX13, which are present in both *stx*-positive and *stx*-negative disease-linked isolates closely related to the O104:H4 outbreak strain. We found non-O104:H4 ST678 strains from a variety of countries in Europe, Africa, and the Americas, and several of the cases were associated with travel to Africa and Asia, suggesting that these virulence-associated strains are globally distributed. Like the O104:H4 outbreak strain, the AAF/I-positive ST678 strains had similar chromosomes, similar pAA-linked virulence genes, as well as similar virulence factors, including the SPATEs (5, 7, 8, 12). The most salient difference in the chromosomes of these strains from that of the outbreak strain was their respective OAGCs and other mobile genetic elements. Thus, the horizontal exchange of these clusters appears to have been a critical step in the evolution of these new pathogenic serotypes, some of which were linked to HUS or bloody diarrhea.

The prime example uncovered here is the likely derivation of O181:H4 pathogens from an O104:H4 precursor via OAG exchange. Among the 21 O181:H4 strains, 6 harbored an *stx*_2a_-carrying prophage, including HUS-linked strain 17-07187. In four of these strains, the *stx*_2a_ prophage was very similar to the *stx*_2a_ prophage of the 2011 outbreak strain (Fig. 3A), suggesting that the O-antigen exchange was a more recent event in their evolution than the acquisition of the Shiga toxin-encoding prophage. The absence of *stx* prophages in 15 of the O181:H4 isolates may be due to a lack of *stx* prophage acquisition or may have resulted from the loss of their *stx* prophages, which is well documented in STEC isolates of other serotypes (23). Thus, in addition to O-antigen exchange, the ongoing horizontal transmission of mobile genetic elements such as *stx* phages has contributed to the diversification of diarrheagenic ST678.

OAGCs of Gram-negative bacteria are hot spots for diversifying selection and recombination events (15, 19). Serotype conversion by the lateral exchange of OAGCs has played an important role in the evolution of enteric pathogens. For example, Vibrio cholerae serogroup O139, which arose from the exchange of O1 and O139 OAGCs, transiently replaced the dominant O1 group as the cause of cholera from 1992 to 1993 (13, 24). Our discovery of seven distinct O groups that share the flagellar H4 antigen and a highly similar virulence-linked genetic makeup provides a compelling example of the role of O-antigen exchange in the diversification of diarrheagenic E. coli. In addition, STEC O157:H7 is thought to have arisen from an enteropathogenic E. coli (EPEC)-like O55:H7 strain that initially acquired *stx* via phage transduction and subsequently acquired the O157 O group by the exchange of the O55 with the O157 OAGC (25). Also, an O182-O156 switch is thought to have occurred in STEC strains persistently infecting cattle (18). Consequently, different OAGs may be found in highly related genotypes (26, 27).

We can only speculate about the conditions driving OAGC exchange among ST678 strains. Shiga toxin-producing O104:H4 EAEC strains, such as the 2011 outbreak strain, are considered human-restricted pathogens and have not been isolated from animals such as cattle (28). However, interestingly, a recent description highlighted the detection of an STEC O104:H4 strain in pork (29). It is possible that OAGC exchange occurred in a human host where the human intestinal microbiome may contain E. coli strains of O groups such as O181 and O127 that could have donated their OAGC to an O104:H4 recipient by some means of horizontal exchange. Also, epidemiological investigations suggest that fenugreek sprouts were the food source that initiated the STEC O104:H4 epidemic; therefore, plants colonized by microbiota may be another possible site for OAGC exchange (4).

### Conclusion.

In conclusion, our study highlights how the lateral exchange of OAGCs can lead to the rapid diversification of a globally important pathogen. Furthermore, an important clinical implication of these findings is that serotype surveillance cannot be used as a simple proxy for strain virulence and needs to be complemented by virulence gene or genome analysis. Surveillance to uncover how highly virulent strains may reemerge and spread in new O-antigen outfits is warranted.

## MATERIALS AND METHODS

### Study strains.

In the context of intensified STEC surveillance in Germany, clinical isolates were collected at the National Reference Center for Salmonella and Other Bacterial Enteric Pathogens and analyzed for serotype, *stx* and subtypes, *eaeA*, *hlyA*, and *aatA* as described previously (30). Strains were grown on nutrient agar (Oxoid GmbH, Germany), Luria-Bertani (LB) broth, or enterohemolysin agar (Sifin GmbH, Germany). Genome sequencing was carried out on a subset of the strains, and further genome sequence data for the strains were gathered from EnteroBase and the NCBI (see Table S1 in the supplemental material).

### Whole-genome sequencing.

Long-read WGS of O181:H4 strain 17-07187 was performed by GATC Biotech (Constance, Germany) using a Pacific Biosciences (PacBio) RS II sequencer, and short-read genome sequencing of strains 12-04810, 14-03615, 14-01288, 16-00596, 16-01499, 16-05332, 17-01774, 17-00416, 17-07187, and 19-02696 (Table S1) was performed on Illumina MiSeq and HiSeq 1500 benchtop sequencers.

### Bioinformatics analyses.

The *de novo* assembly of the PacBio sequence data (103-fold average coverage) was performed by GATC Biotech by utilizing HGAP3 (Pacific Biosciences, USA). Polishing of the assembled genome and plasmids was performed with Illumina short reads by using Pilon (version 1.22) (31). Quality control and trimming of MiSeq raw reads with the subsequent detection of the serotype and virulence genes were performed as described previously (16). Genomic comparisons were carried out using MAUVE (version 1.1.1) and MAFFT (version 1.3.7) as plug-ins in Geneious (version 11.1.5; Biomatters Ltd.) (32, 33). Gaps within the MAFFT alignment were excluded using the Geneious mask alignment function. Ridom SeqSphere+ (version 7.2.0; Ridom GmbH, Germany) was used to determine MLST Warwick sequence types and to create minimum-spanning trees based on 2,513 allele targets (here, genes) from the E. coli cgMLST EnteroBase scheme with pairwise ignoring of missing values from genome assemblies (20). Different variants of the cgMLST genes among different genomes are represented as ADs. Phage prediction was carried out by analyzing the genome sequences using PHASTER (34). RAST was used for coding sequence (CDS) annotation (35).

### SNP-based alignment and maximum likelihood-based phylogenetic tree.

The mapping of sequencing reads, the generation of consensus sequences, and alignment calculations were performed using the BatchMap pipeline with the FWSEC0009 genome sequence as a reference (36). Gubbins (version 3.2.1) was used to identify loci containing elevated densities of base substitutions (putative recombinations) within the alignment while concurrently constructing an alignment and phylogeny (RAxML tree) based on the putative point mutations (SNPs) outside these regions (37). The alignment generated by Gubbins was used to create a maximum likelihood-based phylogenetic tree using PhyML 3.3.20180214 (Geneious plug-in, substitution model HKY85, and 100 bootstraps) (38).

### Temporal signal and “clocklikeness” of molecular phylogenies.

TempEst was used to analyze the RAxML tree generated by Gubbins in conjunction with collection year data to validate the molecular-clock assumption (39). The best-fitting root was chosen for linear regression analyses.

### Cytotoxicity, adherence, and infection assays.

The viability of Vero cells after inoculation with diluted bacterial culture supernatants (1:200) was examined using 3-(4,5-dimethylthiazole-2-yl)-2,5-diphenyltetrazolium bromide (MTT) (5). Bacteria and Vero cells were prepared as described previously (30). Adherence to HEp-2 cells was performed as described previously (36). For infant rabbit infection assays, litters of mixed-gender 2-day-old New Zealand White infant rabbits with the lactating doe were acquired from Charles River Canada (strain code 052). Infant rabbits were orogastrically inoculated on the day of arrival with 10^9^ CFU of streptomycin-resistant O104:H4 strain C227-11 and O181:H4 strain 17-07187 suspended in 500 μL of 2.5% sodium bicarbonate (pH 9) using a size 4 French catheter as described previously, except that no ranitidine was administered (12). No antibiotics were used prior to or during infection. Infant rabbits were monitored for signs of illness and euthanized at 3 days (68 to 72 h) postinfection. Tissue samples taken from the stomach, small intestine, cecum, and colon were homogenized in sterile phosphate-buffered saline (PBS) using a minibeadbeater-16 instrument (Biospec Products, Inc.), and CFU were determined by serial dilution and plating onto LB medium containing 200 μg/mL streptomycin (12).

### Ethics statement.

Rabbit studies were conducted according to protocols reviewed and approved by the Brigham and Women’s Hospital Committee on Animals (IACUC protocol 2016N000334) and Animal Welfare Assurance of Compliance (number A4752-01) in accordance with recommendations in the *Guide for the Care and Use of Laboratory Animals* of the National Institutes of Health (40) and the Animal Welfare Act of the U.S. Department of Agriculture.

### Data availability.

The generated sequences were uploaded to the NCBI database under BioProject accession number PRJNA833419.
