# Supplementary material for: O-Antigen Diversification Masks Identification of Highly Pathogenic Shiga Toxin-Producing Escherichia coli O104:H4-Like Strains
Source: Microbiol Spectr. 2023 May 22;11(3):e00987-23. doi: 10.1128/spectrum.00987-23 (PMC10269612; doi:10.1128/spectrum.00987-23)
Supplement: Supplemental file 1 — Supplemental material. Download spectrum.00987-23-s0001.pdf, PDF file, 1.2 MB [file spectrum.00987-23-s0001.pdf]

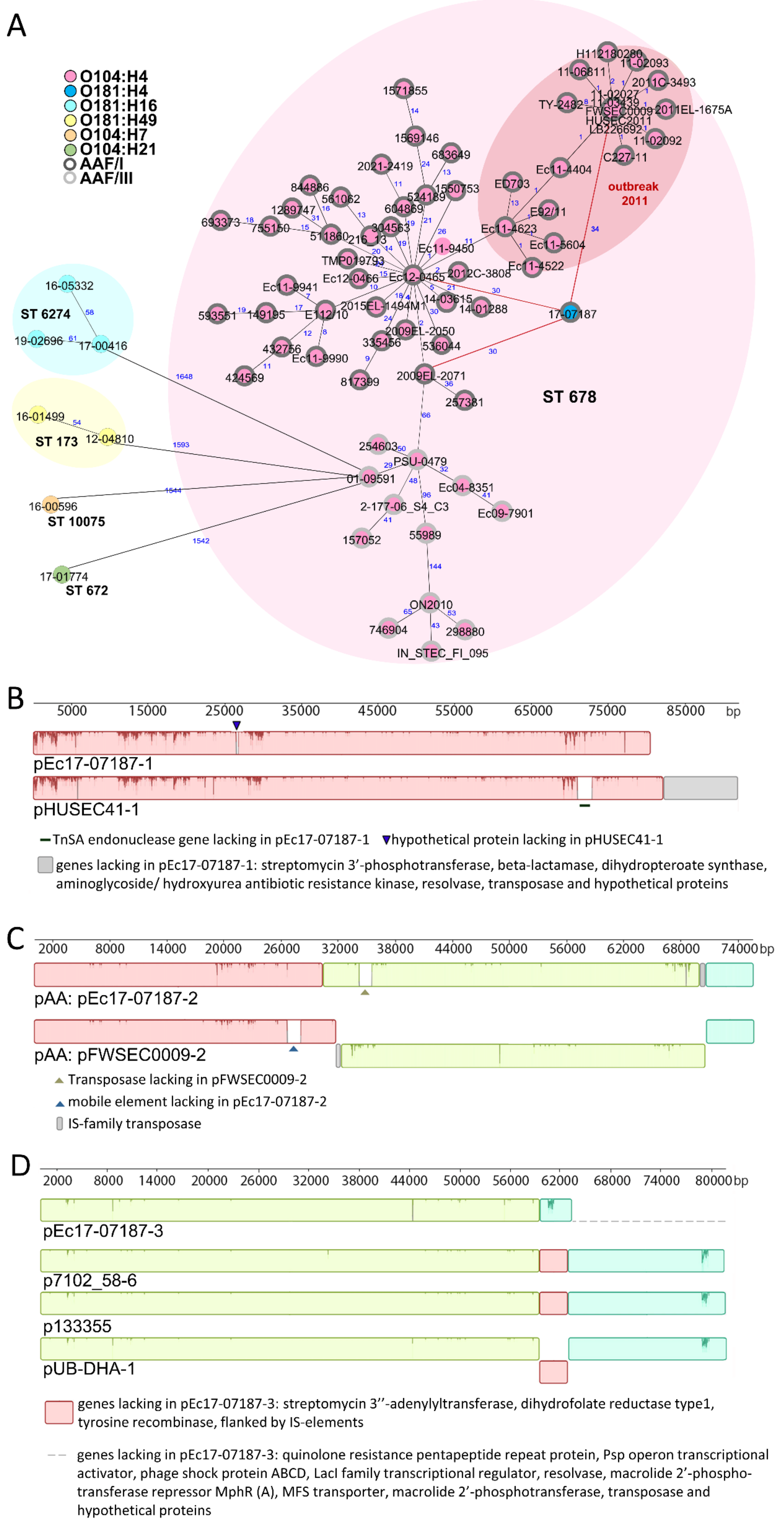

Figure S1: (A) Minimal spanning tree of O181:H4 17-07187 and selected O104:H4 strains based on cgMLST involving 2513 alleles confirms their close phylogenetic relationship. Strains shown in Fig. 1B representing phylogenetic diversity of O104:H4 isolates from humans with respect to isolation time (from 1998 to 2022) and location were selected. Only one cluster representative was used, except of the 2011 outbreak. An allele represents a variant form of a gene. Numbers in blue indicate allelic distances. (B-D) The O181:H4 STEC strain 17-07187 and the O104:H4 outbreak strain harbor a partially distinct set of plasmids with overlapping pAA. MAUVE alignments highlight that (B) STEC O181:H4 17-07187 plasmid 1 pEc17-07187-1 is most similar to pHUSEC41-1 of STEC O104:H4 HUSEC41 from 2001 but lacks antibiotic resistance genes, (C) O181:H4 17-07187 plasmid 2 pEc17-07187-2 is very similar to pAA of the O104:H4 outbreak strain, and (D) O181:H4 17-07187 plasmid 3 pEc17-07187-3 is similar to DHA-1 plasmids of enterobacteria but lacks antibiotic resistance genes.

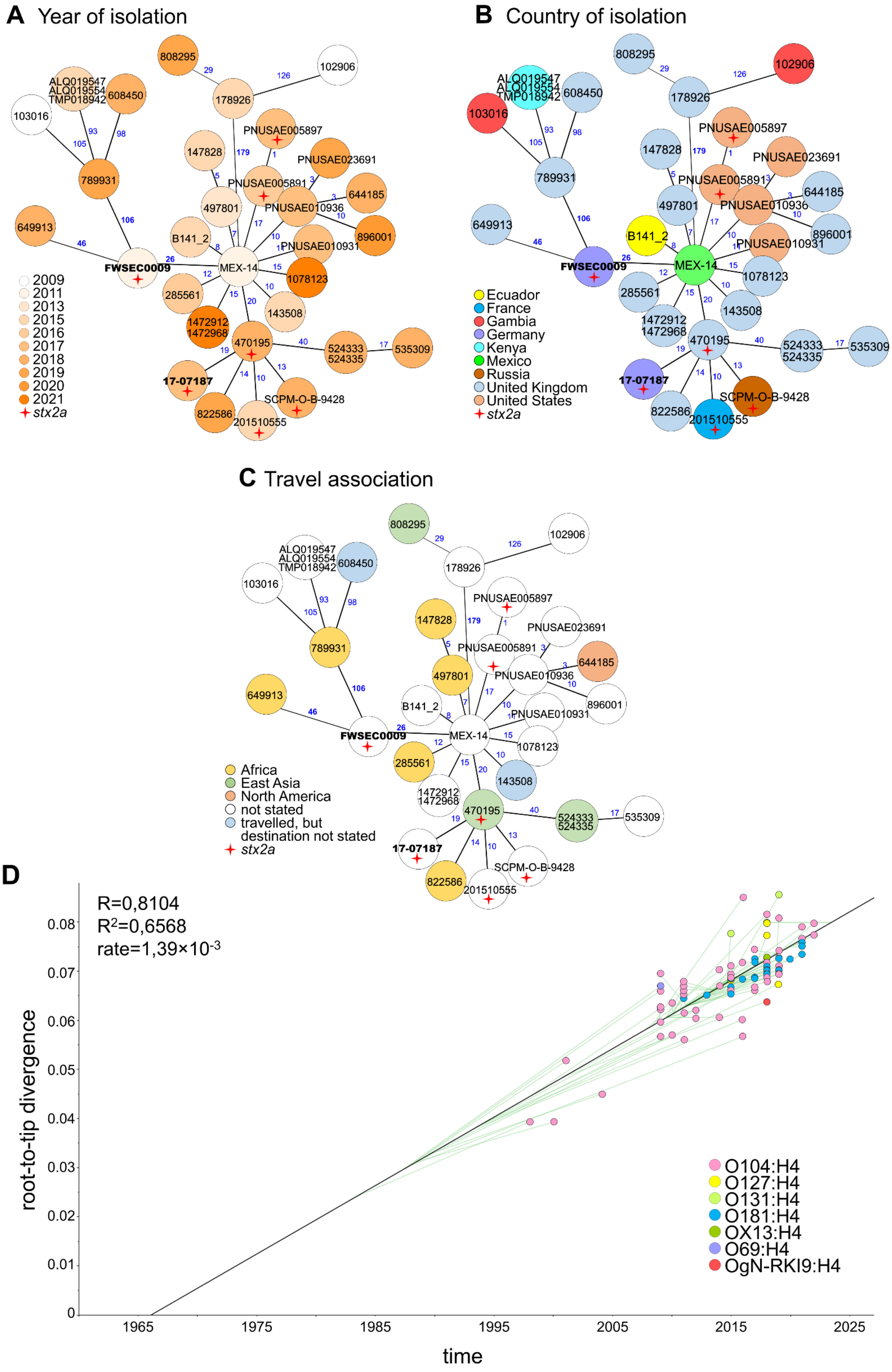

Figure S2: Minimal spanning tree based on cgMLST of the 34 non-O104:H4 ST678 strains and the O104:H4 outbreak strain FWSEC0009 colored by year of isolation (A), country of isolation (B), and travel association (C). Numbers in blue indicate allelic distances. TempEst revealed a positive correlation (R=0.81; R^2=0,66) between isolation time and genetic divergence represented by the root to tip regression analysis using RAxML tree generated by Gubbins based on a recombination-corrected alignment of genome wide polymorphic sites with O104:H4 2011 outbreak strain FWSEC0009 as reference (D). Dots represent the different strains colored by serotype.

Table S2: Genome characteristics of HUS-associated STEC O181:H4 17-07187 and the 2011 STEC O104:H4 outbreak strain FWSEC0009. CDS = coding sequences: determined by means of RAST annotation (http://rast.nmpdr.org/rast.cgi)

**Strain**	**17-07187**	**FWSEC0009**
**Serotype**	**O181:H4**	**O104:H4**
**MLST**	ST678	ST678
**Epidemiology**	Sporadic case	2011 EHEC outbreak
**Year/Country of isolation**	2017 / Germany	2011 / Germany
**Clinics**	HUS	Not stated
**Accession**	NCBI: project: PRJNA833419	NCBI: CP031902
**chromosome size**	5 155 820 bp	5 277 234 bp
**GC content%**	50.7	50.7
**chromosome CDS[#]**	5137	5270
**plasmid 1**	80 851 bp	88 545 bp
**plasmid 1 CDS**	117	114
**plasmid 2/pAA**	75 597 bp	74 217 bp
**plasmid 2/pAA CDS**	128	126
**plasmid 3**	63 387 bp	1 549 bp
**plasmid 3 CDS**	96	3
